# Tendency of K562 Chronic Myeloid Leukemia Cells Towards Cell Reprogramming

**DOI:** 10.4274/tjh.2018.0106

**Published:** 2018-11-13

**Authors:** Açelya Yılmazer Aktuna

**Affiliations:** 1 Ankara University Faculty of Engineering, Department of Biomedical Engineering, Ankara, Turkey

**Keywords:** Induced pluripotent stem cells, Chronic myeloid leukemia, K562, Disease modeling, Cell reprogramming

## Abstract

**Objective:**

Cancer cell reprogramming is a potential tool to study cancer progression, disease pathology, and drug sensitivity. Prior to performing cancer reprogramming studies, it is important to evaluate the stemness predisposition of cells that will be reprogrammed. We performed a proof-of-concept study with chronic myeloid leukemia K562 cells in order to evaluate their tendency for cancer cell reprogramming.

**Materials and Methods:**

Expression of reprogramming factors, pluripotency markers, and tumor-suppressor genes was analyzed at gene and protein levels via real-time reverse transcription-polymerase chain reaction and flow cytometry. Human peripheral blood mononuclear cells (PBMCs) were used as a positive control.

**Results:**

K562 cells were shown to express higher levels of most of the reprogramming factors and pluripotency markers. Expression of p53, which is one of the main regulators during the generation of induced pluripotent stem cells, was found to be lower in K562 cells compared to PBMCs, whereas the other tumor-suppressor genes showed higher expression levels.

**Conclusion:**

This study suggested that, similar to healthy human PBMCs, K526 cells could be used in cancer cell reprogramming studies. Generating induced pluripotent stem cells from leukemia cells could help scientists to establish chronic myeloid leukemia models in vitro for a better understanding of therapy resistance and development of novel therapeutic targets.

## Abstract (tr)

**Amaç:**

Kanser hücrelerinin yeniden programlanarak hastalık modellerinin oluşturulması, hastalığın ilerleyişini, patolojisini ve ilaç duyarlılığını incelemek için önemli bir teknolojidir. Kanser hücrelerinde yeniden programlama çalışmalarını gerçekleştirmeden önce, hücrelerin programlamaya yatkınlığını değerlendirmek önemlidir. Kronik myeloid lösemi K562 hücrelerinin kanser programlama çalışmalarında kullanılabilirliğini göstermek amacıyla bir kavram kanıtı çalışması gerçekleştirdik.

**Gereç ve Yöntemler:**

Yeniden programlama faktörleri, pluripotensi belirteçleri ve tümör baskılayıcı genlerin ifadeleri, gerçek zamanlı-polimeraz zincir reaksiyonu ve akan hücre sitometrisi ile gen ve protein seviyelerinde analiz edildi. Programlama çalışmalarında en çok kullanılan insan periferik kan mononükleer hücreleri (PBMC) pozitif kontrol olarak kullanıldı.

**Bulgular:**

K562 hücrelerinin, yeniden programlama faktörlerini ve pluripotency belirteçlerini PBMC hücrelerine göre daha yüksek seviyede ifade ettiği gösterilmiştir. Uyarılmış pluripotent kök hücrelerinin oluşumu sırasında ana düzenleyicilerden biri olan p53’ün ifadesi, K562 hücrelerinde PBMC’ye kıyasla daha düşük bulunurken, diğer tümör baskılayıcı genler daha yüksek ifade göstermiştir.

**Sonuç:**

Bu çalışma, sağlıklı insan PBMC’lerine benzer şekilde, K526 hücrelerinin yeniden programlama çalışmalarında kullanılabileceğini göstermiştir. Lösemi kaynaklı uyarılmış pluripotent kök hücreleri kullanılarak in vitro ortamda hastalık modellerinin üretilmesi, bilim insanlarının ilaç dirençlerini daha iyi anlaması ve yeni tedavi hedefleri geliştirilmesi için önemli bir araç olacaktır.

## Introduction

Since their discovery, induced pluripotent stem cells (iPSCs) have been extensively used to model diseases and test drugs in vitro [1,2,3,4,5,6]. Hematological disorders including chronic myeloid leukemia (CML) have been modeled by various research groups [4,7,8]. iPSCs capture the genetic alterations present in leukemia cells and their differentiation ability helps scientists to understand disease progression and therapy resistance. 

In one of the first such studies, the CML cell line KBM7 was reprogrammed towards pluripotency via retroviral vectors carrying OKSM (Oct3/4, Klf-4, Sox-2, c-Myc) factors [9]. Unlike the untreated cells, the reprogrammed group showed resistance to the chemotherapeutic agent imatinib, which is an inhibitor of the BCR-ABL oncogene. It was hypothesized that the therapeutic agent imatinib targets cells in a specific epigenetic differentiated cell state, which can contribute to its inability to fully eradicate disease in CML patients [10]. Later, Bedel et al. [11] reported that when CD34^BCR-ABL+^ cells from CML patients were reprogrammed, CML-iPSCs lost their BCR-ABL dependency and became resistant to tyrosine kinase inhibitor therapy. The authors suggested that CML-iPSCs can be used to study mechanisms by which leukemic stem cells survive to therapy and are a promising tool for testing and screening new therapeutic targets reducing leukemic stem cell survival [11]. In another CML study, again with the use of retroviral vectors, iPSCs were generated from primary CML patients’ cells. Although CML-iPSCs were resistant to the chemotherapeutic agent imatinib, CML-iPSC-derived hematopoietic cells recovered sensitivity to the drug [12]. In another study, whole-genome sequencing of CML-derived iPSCs revealed genocopying of highly mutated primary leukemic cells, which were used to understand the selective growth under tyrosine kinase inhibitor therapies [13]. In 2015, iPSCs were used to identify the leukemia stem cells for primitive CML by Suknuntha et al. [14]. Due to the rarity of leukemia stem cells within the primitive hematopoietic cell compartment, it is difficult to study their contribution. By the generation of CML-iPSCs, the authors discovered olfactomedin 4 as a novel factor that contributes to the survival and growth of somatic lin(-)CD34(+) cells from the bone marrow of patients with CML in the chronic phase, but not primitive hematopoietic cells from normal bone marrow [14]. These contradictory results show that more work is needed to model CML. However, as in the reprogramming of healthy cells, there are various factors affecting reprogramming efficiency, and for this reason, these factors should be first determined for leukemia in order to model such diseases in vitro.

As can be seen from the above studies, reprogramming cancer cells is a potential tool to study cancer progression, disease pathology, and drug sensitivity. Prior to performing cancer reprogramming studies, it is important to evaluate the stemness predisposition of cells that will be reprogrammed [4]. Here, we performed a proof-of-concept study with K562 cells in order to evaluate their tendency for cancer cell reprogramming. We analyzed the endogenous expression of reprogramming and pluripotency factors that are known to be important factors for cell reprogramming. Furthermore, it is well known that the expression of tumor-suppressor genes also determines reprogramming efficiency [15]. Therefore, the levels of important tumor-suppressor genes were identified in K562 cells.

## Materials and Methods

### Cell Culture

Human CML cell line K562 and human peripheral blood mononuclear cells (PBMCs) were obtained from ATCC and Lonza, respectively. Cells were cultured in RPMI 1640 supplemented with 10% fetal bovine serum, 50 U/mL penicillin, 50 µg/mL streptomycin, and 1% L‐glutamine at 37 °C in 5% CO_2_.

### RNA Extraction and Quantitative Real-Time PCR (qRT-PCR)

Cells (1x10^6^ cells) were collected and RNA was extracted with the Macherey-Nagel RNA isolation kit. cDNA synthesis from 1 µg of RNA sample was performed with the iScript cDNA synthesis kit (Bio-Rad) according to the manufacturer’s instructions. Two microliters of each cDNA sample were used to perform real-time quantitative reverse transcription-polymerase chain reaction (qRT-PCR) reactions with iQ SYBR Green SuperMix (Bio-Rad). Samples were run on the CFX-96 Connect Real-Time System (Bio-Rad) with the following protocol: 95 °C for 3 min, 1 cycle; 95 °C for 10 s and 60 °C for 30 s, repeated for 40 cycles. GAPDH was used as a reference gene and gene expression levels for OCT3/4, SOX2, KLF4, CMYC, NANOG, REX, CRIPTO, P53, P21, P16, and PRB were normalized to PBMCs.

### Flow Cytometry Analysis

Cells (1x10^6^ cells) were collected by centrifugation, washed with ice-cold methanol for fixation, and then permeabilized with 0.1% Triton-X100 containing 2% bovine serum albumin-phosphate buffered saline (PBS). Following washing with PBS, cells were stained with rabbit anti-Oct3/4, rabbit anti-Nanog, or mouse anti-p53 antibodies. Anti-rabbit-AF543 and anti-mouse-AF488 were used as secondary antibodies. Cells were analyzed with a BD Accuri Plus Flow Cytometer (BD). For 10,000 events, percentages of positive populations were determined by using BD Accuri Plus software (BD).

### Statistical Analysis

Triplicates containing required amounts of cells were used during analyses. Delta Ct values were used for the statistical analysis of RT-PCR results. Statistical analysis was performed by analysis of variance and Tukey’s pairwise comparisons using SPSS 16.0 (SPSS Inc.).

## Results

In order to test the tendency of K562 cells towards cell reprogramming, we used human PBMCs as a positive control because there are studies that have shown successful reprogramming with PBMCs [16].

**Figure 1:**
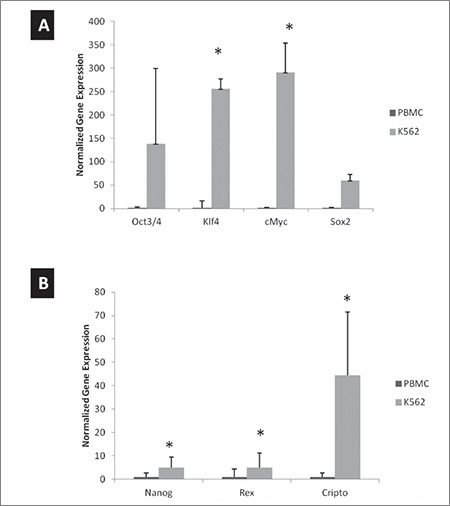
Gene expression of reprogramming factors and pluripotency markers. RNA was isolated from peripheral blood mononuclear cells (PBMCs) and K562 cells and quantitative real-time polymerase chain reaction was performed. Relative gene expression was plotted for A) reprogramming factors and B) pluripotency markers. GAPDH was used as a reference gene and data were normalized to PBMCs. *p<0.05 compared to PBMCs.

As shown in Figure 1, the reprogramming factors (Oct3/4, Klf2, Sox2, cMyc) were all upregulated in K562 cells compared to PBMCs (Figure 1A). Significant differences were observed for the Klf2, Sox2, and cMyc genes. When the cells were analyzed for the expression of pluripotency markers including Nanog, Rex, and Cripto via real-time RT-PCR, we observed higher expression levels compared to PBMCs (Figure 1B). However, we obtained a lower profile when compared to that of the programming factors. 

**Figure 2:**
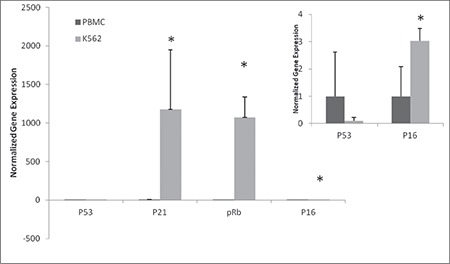
Gene expression of tumor-suppressor genes. RNA was isolated from peripheral blood mononuclear cells (PBMCs) and K562 cells and quantitative real-time polymerase chain reaction was performed. Relative gene expression was plotted for tumor-suppressor genes. GAPDH was used a reference gene and data were normalized to PBMCs. *p<0.05 compared to PBMCs. Small graph shows the gene expression profile of p53 and p16 in order to better represent the differences.

In addition to the programming and pluripotency factors, the expression of tumor-suppressor genes determines the efficiency of reprogramming [15]. For this reason, we analyzed the expression of the P53, P21, P16, and PRB genes and found that P53 was downregulated in K562 cells compared to PBMCs, whereas the others showed higher expression levels (Figure 2). 

**Figure 3:**
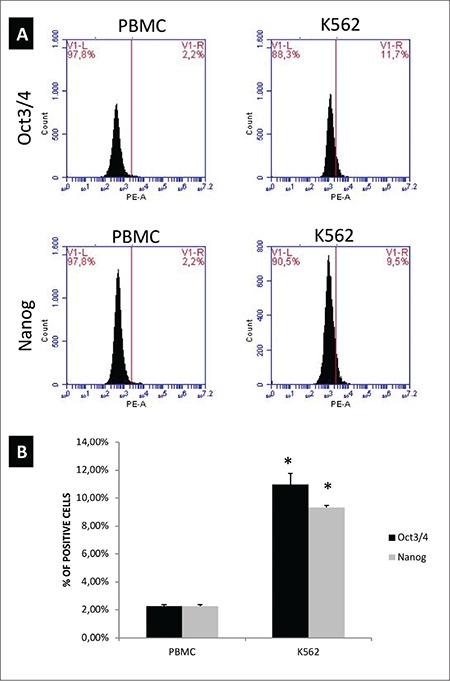
Protein expression of reprogramming factor Oct3/4 and pluripotency marker Nanog. Cells were collected by centrifugation, followed by staining for Oct3/4 and Nanog. Cells were analyzed in a BD Accuri Plus Flow Cytometer (BD). *p<0.05 compared to peripheral blood mononuclear cells. PBMC: Peripheral blood mononuclear cell.

**Figure 4:**
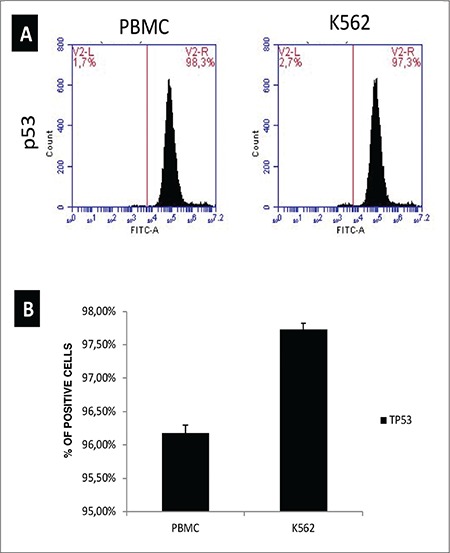
Protein expression of tumor-suppressor gene p53. Cells were collected by centrifugation, followed by staining for p53. Cells were analyzed in a BD Accuri Plus Flow Cytometer (BD). PBMC: Peripheral blood mononuclear cell.

In order to confirm the gene expression data, we performed flow cytometric analysis of programming factor Oct3/4, pluripotency marker Nanog, and tumor suppressor p53 in PBMCs and K562 cells. Flow cytometry analyses confirmed the real-time RT-PCR data. The percentage of cells positive for Oct3/4 and Nanog was increased to 11.7% and 9.5%, respectively (Figure 3). When anti-P53 antibodies were used to stain the cells in flow cytometry, in contrast to real-time RT-PCR data, there was no significant change in the P53-positive cell populations (Figure 4). This may suggest that even though there was a difference at the mRNA level, protein levels do not vary, possibly due to posttranscriptional regulation.

## Discussion

It has been previously reported that the expressions of reprogramming and pluripotency factors in the starting cells are limiting factors in cell reprogramming [4,17]. As shown here, higher levels of reprogramming and pluripotent factors at both gene and protein levels increase the tendency towards cellular reprogramming. On the other hand, expression of tumor-suppressor genes needs to be controlled during iPSC generation [15,18,19]. In this study, we observed downregulation of P53 mRNA and similar levels of its protein in K562 cells compared to PBMCs. However, the overall expressions of other tumor-suppressor genes can still be limiting factors. Therefore, the expression of these genes should be carefully monitored during reprogramming. Silencing strategies could be needed to achieve efficient reprogramming. Until now, cancer reprogramming studies for CML have not focused on the expression of the above factors [9,11,13,20,21] and there is no study that has reported the link between these factors and the ease of reprogramming. Therefore, this is an important preliminary study that reinforces the importance of these factors.

Screening the levels of reprogramming and pluripotency factors has been one of the ways to assess the efficiency of iPSC generation [4]. On the other hand, expression of these markers has also been linked with multidrug resistance of leukemia cells through modulating the ATP-binding-cassette transporters (ABC-transporters) [9,21]. For example, the expressions of Oct4, Sox2, and Nanog, all of which are studied in the present work, have been shown to be upregulated in K562 cells when they retain multidrug resistance to doxorubicin [20,22]. Therefore, this also suggests that monitoring their expression status is a key step in order to model the disease and study drug resistance.

In addition to the above factors, there are other factors that limit the efficiency of iPSC generation from cancer cells. These include the proliferation rate of cancer cells, the epigenetic background, long-term culturing conditions during reprogramming, the heterogeneity of tumor cells, and the presence of cancer stem cells [4]. For example, highly proliferating cells will be difficult to reprogram since dividing cancer cells and reprogrammed cancer cells will compete in culture conditions, which would not allow for ground-state pluripotency in the reprogrammed cells [23]. Therefore, future studies should monitor the above parameters in detail during cell reprogramming of CML.

Considering PBMC usage as the cell source in reprogramming protocols, this proof-of-concept study showed that K526 CML cells could also be used in cancer cell reprogramming studies. Generating iPSCs from these cell lines could help scientists to establish CML models in vitro. This can allow us to study disease progression, drug responses, and disease pathology.

## Conclusion

This study suggested that, similar to healthy human PBMCs, K526 cells could be used in cancer cell reprogramming studies. Generating iPSCs from leukemia cells could help scientists to establish CML models in vitro, better understand disease progression, and develop novel therapeutic targets.
